# Nationwide population-based cohort study of psychiatric disorders in individuals with Ehlers–Danlos syndrome or hypermobility syndrome and their siblings

**DOI:** 10.1186/s12888-016-0922-6

**Published:** 2016-07-04

**Authors:** Martin Cederlöf, Henrik Larsson, Paul Lichtenstein, Catarina Almqvist, Eva Serlachius, Jonas F. Ludvigsson

**Affiliations:** Department Medical Epidemiology and Biostatistics, Karolinska Institutet, Stockholm, 17177 Sweden; Astrid Lindgren Children’s Hospital, Karolinska University Hospital, Stockholm, Sweden; Department of Clinical Neuroscience, Karolinska Institutet, Centre for Psychiatry Research, Stockholm Health Care Services, Stockholm County Council, Stockholm, Sweden; Department of Pediatrics, Örebro University Hospital, Örebro University, Örebro, Sweden; Division of Epidemiology and Public Health, School of Medicine, University of Nottingham, Nottingham, UK; Department of Medicine, Columbia University College of Physicians and Surgeons, New York, NY USA

**Keywords:** Cohort study, Ehlers-Danlos syndrome, Hypermobility syndrome, Epidemiology, Psychiatric disorders

## Abstract

**Background:**

To assess the risk of psychiatric disorders in Ehlers-Danlos syndrome (EDS) and hypermobility syndrome.

**Methods:**

Nationwide population-based matched cohort study. EDS, hypermobility syndrome and psychiatric disorders were identified through Swedish national registries. Individuals with EDS (*n* = 1,771) were matched with comparison individuals (*n* = 17,710). Further, siblings to individuals with EDS who did not have an EDS diagnosis themselves were compared with matched comparison siblings. Using conditional logistic regression, risk of autism spectrum disorder (ASD), bipolar disorder, attention deficit hyperactivity disorder (ADHD), depression, attempted suicide, suicide and schizophrenia were estimated. The same analyses were conducted in individuals with hypermobility syndrome (*n* = 10,019) and their siblings.

**Results:**

EDS was associated with ASD: risk ratio (RR) 7.4, 95 % confidence interval (95 % CI) 5.2–10.7; bipolar disorder: RR 2.7, CI 1.5–4.7; ADHD: RR 5.6, CI 4.2–7.4; depression: RR 3.4, 95 % CI 2.9–4.1; and attempted suicide: RR 2.1, 95 % CI 1.7–2.7, but not with suicide or schizophrenia. EDS siblings were at increased risk of ADHD: RR 2.1, 95 % CI 1.4–3.3; depression: RR 1.5, 95 % CI 1.1–1.8; and suicide attempt: RR 1.8, 95 % CI 1.4–2.3. Similar results were observed for individuals with hypermobility syndrome and their siblings.

**Conclusions:**

Individuals with EDS and hypermobility syndrome are at increased risks of being diagnosed with psychiatric disorders. These risk increases may have a genetic and/or early environmental background as suggested by evidence showing that siblings to patients have elevated risks of certain psychiatric disorders.

**Electronic supplementary material:**

The online version of this article (doi:10.1186/s12888-016-0922-6) contains supplementary material, which is available to authorized users.

## Background

Ehlers-Danlos syndrome (EDS) is the name applied to a rather large group of inherited disorders that affect the connective tissue, the tissue that provides support to many parts of the body. This genetic disorder is present in at least 1/5000 individuals [1] and an important cause of joint hypermobility syndrome [2]. The disease is characterized by hypermobility, although symptoms and signs can be highly variable and include joint complaints, myalgia, skin problems, sleep apnea, pneumothorax and cardiovascular disease. While a diagnosis of EDS can be comforting to the patient, there is no curative treatment for EDS. Instead, physicians aim to stabilize joints and prevent complications, sometimes using surgery to resolve the condition. Physiotherapy is an integral part of patient management [3].

Intense pain, diagnostic delay and risk of unemployment due to musculoskeletal complications, all contribute to the emotional burden of the disorder [4]. Already in 1994, Lumley et al*.* reported a link between EDS and psychological problems [5]. Despite the long-term awareness of psychosocial dysfunctioning in EDS, studies on psychiatric disorders in EDS or hypermobility syndrome are rare, but well summarized in a recent meta-analysis by Smith et al*.* [4]. Smith et al*.* found a fourfold increased risk of both anxiety and depression in hypermobility syndrome, while the risk of panic disorder was even higher (odds ratio (OR) = 6.7, 95 % confidence interval (95 % CI) 2.2–20.4). However, the OR for anxiety was based on only 253 patients with joint hypermobility and the three studies [6–8] contributing data on anxiety found substantially different risk estimates with the most recent paper reporting a lack of association but with wide CIs (OR = 0.9, 95 % CI 0.1–5.6) [7]. Examining anxiety scores, levels were higher in the 501 individuals with joint hypermobility (standardized mean difference in score: +0.5) than in the 948 controls [4].

Further, other psychiatric disorders have been implicated in EDS [9]. Depression [6, 7, 10] has been linked to joint hypermobility (OR 4.1, 95 % CI 1.8–9.4) [4], but studies have been small and study participants may not have reflected the average patient with EDS or hypermobility syndrome. One study found a striking occurrence of depression in EDS (69 % of the patients had a record of depression) [11].

While we are unaware of any research in EDS and schizophrenia, there are several case reports of an association between the EDS-like Marfan Syndrome and schizophrenia [12]. Similarly, there are case reports of EDS and autism spectrum disorders (ASDs) [4, 13], but to our knowledge no case–control or cohort studies exist on the topic.

Given the inconsistent and sometimes flawed data on EDS, hypermobility syndrome and psychiatric disorder, we decided to conduct a population-based matched cohort study examining ASD, bipolar disorder, ADHD, depression, suicide attempt, completed suicide and schizophrenia in patients with EDS or hypermobility syndrome. We also examined the same psychiatric disorders in siblings to patients with EDS or hypermobility syndrome. If both EDS/hypermobility patients and their siblings are at increased risk of psychiatric disorders this suggests that genetic and/or early environmental factors influence the association with psychiatric disorders.

## Methods

Using the Swedish Patient Registry [14], we identified individuals with EDS and hypermobility syndrome. Moreover, through the Multi-Generation Registry we identified siblings to patients with EDS and hypermobility syndrome and their respective comparison siblings.

### EDS and hypermobility syndrome

Individuals with EDS were defined according to the relevant international classification of disease (ICD-10) code in the Swedish Patient Registry (Q79.6). We also examined hypermobility syndrome (ICD-10: M35.7). The Swedish Patient Registry started in 1964 and became nationwide in 1987. Only in 1997 with the introduction of ICD-10 (as opposed to ICD-9, which was used between 1987 and 1996) were there specific codes for EDS and hypermobility syndrome. While we are unaware of any validation of these two diagnoses, most chronic diagnoses have a positive predictive value of 85–95 % in the Patient Registry [14]. Only the first record of either EDS or hypermobility syndrome was used and hence double entries were removed.

### Psychiatric disorders

The outcome measures were the following psychiatric disorders: bipolar disorder (defined according to a validated algorithm [15], ASD (ICD-10: F84), ADHD (ICD-10: F90), depression (ICD-8: 296.0, 300.4, ICD-9: 296B, 300E, 311, ICD-10: F32-33, F34.1, F34.8-9, F38.1), schizophrenia (ICD-8: 295.0-6, 295.8-9, ICD-9: 295A-G, 295W, 295X; ICD-10: F20), suicide attempt (ICD-10: X60-84, Y10-34) and completed suicide (defined via the Cause of Death Registry). ASD was also identified via the Clinical Registry for Child and Adolescent Psychiatry in Stockholm County [16] (ICD-10: F84 and DSM-IV: 299). This latter registry was also used for ADHD (ICD-10: F90 and DSM-IV: 314). To maximize coverage, we employed the Prescribed Drug Registry for ADHD in which cases were defined through treatment with stimulant (amphetamine, dexamphetamine, methylphenidate) or non-stimulant medication (atomoxetine).

Psychiatric disorders have been recorded in the Patient Registry since 1973 [14]. Most validation studies of psychiatric disorders in the Patient Registry have concerned schizophrenia, frequently indicating a positive predictive value of about 90 % [17–19]. Although carried out in neighboring Denmark, a recent validation of ASD in 499 patients found that 469 fulfilled stipulated diagnostic criteria (equal to a positive predictive value of 94.3 %) [20]. The Patient Registry is maintained by the Swedish National Board of Health and Welfare (http://www.socialstyrelsen.se/english). This registry has recorded inpatient care since 1964 (with psychiatric care added in 1973) [14]. Diseases are coded using ICD codes and for each healthcare contact several items are recorded, including personal identity number (PIN) [21], date of admission and discharge, hospital and primary and secondary diagnoses. Through the unique PIN, information on each individual patient can be linked to other registries. Additional file 1: Table S1 outlines the number of patients with each included outcome by the respective registries used for identification.

### Study participants

Each individual with EDS or hypermobility syndrome was matched with up to 10 comparison individuals for same sex, age and county of residence at the year of first diagnosis. Comparison individuals were retrieved from the Total Population Registry [22]. Through this register, we also identified all siblings to our EDS and hypermobility syndrome patients (hereafter referred to as “EDS siblings” and “hypermobility syndrome siblings”, respectively) and all siblings to comparison individuals without EDS and hypermobility syndrome, matched on birth year and sex (hereafter referred to as “comparison siblings”). The part of the Total Population Registry containing data on siblings is usually called “The Multi-Generation Registry”. This Registry contains data on 97 % of the mothers and 95 % of the fathers of index persons. Index persons are restricted to individuals alive on 1 January 1961 and born in 1932 or later [23].

### Statistical analyses

To estimate risks of psychiatric disorders in individuals with EDS or hypermobility syndrome and their siblings, we calculated risk ratios (RR) and 95 % CIs using conditional logistic regression. In the sibling analyses, CIs were obtained with a robust sandwich estimator to adjust for the correlated data structure. In addition, we conducted a secondary analysis restricted to individuals born in Sweden to rule out the possibility that an association with psychiatric disorders would be due to country of birth (as a proxy for ethnicity). In two post-hoc analyses we examined the risk of psychiatric disorders (1) according to age at onset of EDS (≤19 years vs. ≥20 years), and (2) when only considering psychiatric disorders occurring after first EDS diagnosis.

Statistical analyses were conducted in SAS version 9.4 (SAS Institute, Cary, NC, USA)., and the STROBE guidelines for the reporting of observational studies were followed.

## Results

We identified 1,780 individuals with EDS (74 % females), 1,722 EDS siblings, 10,019 individuals with hypermobility syndrome (67 % females) and 11,082 hypermobility syndrome siblings. The vast majority of individuals with EDS (94 %) and hypermobility syndrome (92 %) were born in Sweden. Nine individuals with EDS (0.005 %) were excluded from the analyses because of missing demographic data (remaining: *n* = 1,771). Additional demographic data are given in Additional file 2: Table S2.

### EDS vs. comparison individuals

In all, 52 (2.9 %) EDS patients and 72 (0.4 %) comparison individuals were diagnosed with ASD (RR 7.4, 95 % CI 5.2–10.7, Table 1). Bipolar disorder was diagnosed in 15 (0.9 %) vs. 56 (0.3 %) comparison individuals (RR 2.7, 95 % CI 1.5–4.7), ADHD in 76 (4.3 %) vs. 144 (0.8 %) (RR 5.6, 95 % CI 4.2–7.4), depression in 197 (11.1 %) vs. 643 (3.6 %) (RR 3.4, 95 % CI 2.9–4.1) and 96 (5.4 %) vs. 464 (2.6 %) attempted suicide (RR 2.1, 95 % CI 1.7–2.7). Restricting our data to individuals with EDS that preceded psychiatric disorder, we found positive associations with ASD and ADHD but an inverse relationship with attempted suicide (Additional file 3: Table S3).

**Table 1:** Risks of psychiatric disorders, suicide attempt and suicide in individuals with EDS compared with matched comparison individuals

	EDS individuals (*n* = 1,771)	Matched comparison individuals (*n* = 17,710)		
	*n* (%)	*n* (%)	RR (95 % CI)	RR^a^ (95 % CI)
Autism spectrum disorder	52 (2.9)	72 (0.4)	**7.4 (5.2–10.7)**	**8.4 (5.7–12.4)**
Bipolar disorder	15 (0.9)	56 (0.3)	**2.7 (1.5–4.7)**	**2.6 (1.4–4.7)**
ADHD	76 (4.3)	144 (0.8)	**5.6 (4.2–7.4)**	**5.8 (4.3–7.9)**
Depression	197 (11.1)	643 (3.6)	**3.4 (2.9–4.1)**	**3.5 (2.9–4.2)**
Suicide attempt	96 (5.4)	464 (2.6)	**2.1 (1.7–2.7)**	**2.1 (1.6–2.6)**
Suicide	2 (0.1)	6 (0.03)	3.3 (0.7–16.5)	2.9 (0.6–14.4)
Schizophrenia	3 (0.2)	38 (0.2)	0.8 (0.2–2.6)	0.6 (0.1–2.5)

Associations are expressed as risk ratios (RR) and 95 % confidence intervals (95 % CIs) from conditional logistic regression

Note: statistically significant RR are bolded. RR^a^, risk ratios restricted to individuals born in Sweden

### EDS siblings vs. comparison siblings

ADHD (RR 2.1, 95 % CI 1.4–3.3), depression (RR 1.5, 95 % CI 1.1–1.8) and suicide attempt (RR 1.8, 95 % CI 1.4–2.3) were more frequently diagnosed in siblings to EDS patients than in comparison siblings.

### Hypermobility syndrome vs. comparison individuals

Of the hypermobility syndrome patients, 161 (1.6 %) vs. 1,186 (1.2 %) comparison individuals were diagnosed with ASD (RR 1.4, 95 % CI 1.1–1.6; Table 3). Bipolar disorder was diagnosed in 42 (0.4 %) vs. 296 (0.3 %) comparison individuals (RR 1.4, 95 % CI 1.1–2.0), ADHD in 301 (3.0 %) vs. 546 (0.5 %) (RR 5.8, 95 % CI 5.0–6.7), Depression in 696 (7.0 %) vs. 3,180 (3.2 %) (RR 2.3, 95 % CI 2.1–2.5) and attempted suicide in 423 (4.2 %) vs. 2,121 (2.2 %) (RR 2.1, 95 % CI 1.8–2.3).

### Hypermobility syndrome siblings vs. comparison siblings

ASD (RR 1.3, 95 % CI 1.1–1.7), ADHD (RR 1.3, 95 % CI 1.1–1.5), depression (RR 1.2, 95 % CI 1.1–1.3) and suicide attempt (RR 1.2, 95 % CI 1.1–1.4) were more often diagnosed in hypermobility syndrome siblings as compared with comparison siblings (Table 4).

### Sensitivity analyses

In the analyses restricted to individuals born in Sweden, all associations remained virtually unchanged, except that the association between hypermobility syndrome and bipolar disorder was no longer statistically significant (Tables 2, 3 and 4).

**Table 2:** Risks of psychiatric disorders, suicide attempt and suicide in siblings of individuals with EDS (EDS siblings), compared with matched siblings to individuals without EDS (comparison siblings)

	EDS siblings (*n* = 1,722)	Comparison siblings (*n* = 16,920)		
	*n* (%)	*n* (%)	RR (95 % CI)	RR^a^ (95 % CI)
Autism spectrum disorder	10 (0.6)	56 (0.4)	1.8 (0.9–3.4)	1.7 (0.9–3.2)
Bipolar disorder	7 (0.5)	69 (0.4)	1.0 (0.5–2.0)	0.9 (0.4–2–0)
ADHD	26 (1.5)	123 (0.7)	**2.1 (1.4–3.3)**	**2.1 (1.4–3.3)**
Depression	72 (4.2)	492 (2.9)	**1.5 (1.1–1.8)**	**1.4 (1.1–1.8)**
Suicide attempt	66 (3.8)	364 (2.1)	**1.8 (1.4–2.3)**	**1.8 (1.4–2.3)**
Suicide	10 (0.6)	44 (0.3)	**2.1 (1.2–3.9)**	**2.3 (1.3–4.1)**
Schizophrenia	7 (0.4)	54 (0.3)	1.3 (0.6–2.7)	1.4 (0.6–3.1)

Associations are expressed as risk ratios (RR) and 95 % confidence intervals (95 % CIs) from conditional logistic regression

Note: statistically significant RR are bolded. RR^a^, risk ratios restricted to individuals born in Sweden

**Table 3:** Risks of psychiatric disorders, suicide attempt and suicide in individuals with hypermobility syndrome (including EDS) compared with matched comparison individuals

	Hypermobility syndrome individuals (*n* = 10,019)	Matched comparison individuals (*n* = 100,190)		
	*n* (%)	*n* (%)	RR (95 % CI)	RR^a^ (95 % CI)
Autism spectrum disorder	161 (1.6)	1,186 (1.2)	**1.4 (1.1–1.6)**	**1.3 (1.1–1.6)**
Bipolar disorder	42 (0.4)	296 (0.3)	**1.4 (1.1–2.0)**	1.4 (0.9–1.9)
ADHD	301 (3.0)	546 (0.5)	**5.8 (5.0–6.7)**	**5.5 (4.8–6.4)**
Depression	696 (7.0)	3,180 (3.2)	**2.3 (2.1–2.5)**	**2.3 (2.1–2.5)**
Suicide attempt	423 (4.2)	2,121 (2.2)	**2.1 (1.8–2.3)**	**2.0 (1.8–2.3)**
Suicide	6 (0.1)	3 (0.05)	1.8 (0.8–4.4)	1.9 (0.7–5.0)
Schizophrenia	11 (0.1)	159 (0.2)	0.7 (0.4–1.3)	0.7 (0.4–1.3)

Associations are expressed as risk ratios (RR) and 95 % confidence intervals (95 % CIs) from conditional logistic regression

Note: statistically significant RR are bolded. RR^a^, risk ratios restricted to individuals born in Sweden

**Table 4:** Risks of psychiatric disorders, suicide attempt and suicide in siblings of individuals with hypermobility syndrome (hypermobility syndrome siblings) compared with matched siblings to individuals without hypermobility syndrome (comparison siblings)

	Hypermobility syndrome siblings (*n* = 11,082)	Comparison siblings (*n* = 108,772)		
	*n* (%)	*n* (%)	RR (95 % CI)	RR^a^ (95 % CI)
Autism spectrum disorder	66 (0.6)	500 (0.5)	**1.3 (1.1–1.7)**	**1.3 (1.1–1.7)**
Bipolar disorder	31 (0.3)	269 (0.3)	1.1 (0.8–1.6)	1.2 (0.8–1.7)
ADHD	124 (1.1)	966 (0.9)	**1.3 (1.1–1.5)**	**1.3 (1.1–1.5)**
Depression	343 (3.1)	2,788 (2.6)	**1.2 (1.1–1.3)**	**1.2 (1.1–1.3)**
Suicide attempt	270 (2.4)	2,182 (2.0)	**1.2 (1.1–1.4)**	**1.2 (1.1–1.4)**
Suicide	29 (0.3)	230 (0.2)	1.2 (0.9–1.7)	1.3 (0.9–1.8)
Schizophrenia	26 (0.2)	184 (0.2)	1.4 (0.9–2.0)	1.3 (0.9–1.9)

Associations are expressed as risk ratios (RR) and 95 % confidence intervals (95 % CIs) from conditional logistic regression

Note: statistically significant RR are bolded. RR^a^, risk ratios restricted to individuals born in Sweden

Finally, we found similar risk estimates for psychiatric disorders in EDS patients diagnosed in childhood and adulthood; however, lack of statistical power did not allow estimation of risks of some psychiatric disorders in individuals with EDS aged ≤19 years at their first diagnosis (Additional file 4: Table S4).

## Discussion

In this nationwide population-based cohort study, we examined EDS, hypermobility syndrome and risk of psychiatric disorders. We found a substantially increased risk of several psychiatric disorders in addition to depression [4].

Our cohort of hypermobility syndrome patients was about forty times larger than the total number of hypermobility patients included in a recent meta-analysis [4] (our study included 10,019 individuals with hypermobility syndrome vs. 238 hypermobility patients and depression in Smith et al*.*). The large number of study participants allowed for the calculation of precise risk estimates. Furthermore, our study is the first to examine psychiatric disorders in first-degree relatives to persons with EDS and hypermobility syndrome. This aspect of the study is important in that we were able to demonstrate that familial factors play an important role in the psychiatric comorbidity in EDS and hypermobility syndrome.

### Comparison with the previous literature

Since the 1990s, at least three papers have examined the association between hypermobility syndrome and EDS and anxiety [6–8]. Together, they point towards a significant association between hypermobility syndrome and anxiety. Similarly, some research [6], but not all [7], suggests a positive association with depression. However, most of these studies have been small.

One Spanish study found a 10-fold increased risk of panic disorders in patients with hypermobility syndrome [6]. The same authors recently reported that hypermobility was linked to a 22-fold increased risk of panic/agoraphobia disorders and a 4-fold increase in the use of anxiolytic drugs in patients with hypermobility [10]. However, more than 20 % of the study participants developed hypermobility syndrome [10], limiting the external validity of the findings.

Of note we found a protective effect of earlier EDS on later suicide attempt in one of our post hoc analyses. We believe this is a chance finding although we cannot rule out that some individuals take comfort in receiving a diagnosis (EDS) for which they may have sought help for many years.

Our findings confirm previous evidence of positive associations with psychiatric disorders but also contribute new knowledge. First, our population-based approach maximizes statistical power and minimizes selection bias. In contrast, past research has often been based on patients attending tertiary centers. Such patients generally have a more severe disease than the average patient and this might overestimate risk estimates.

We used the Patient Registry [14] to identify individuals with EDS, hypermobility syndrome and psychiatric disorder. This Registry has been extensively validated with a positive predictive value for most chronic disorders ranging from 85–95 %. However, neither EDS nor hypermobility syndrome has been validated per se in this study. In addition, we lacked information on the Beighton score for hypermobility, presence of pain or number of complaints. Hence, we were unable to predict the risk of psychiatric comorbidity according to EDS or hypermobility phenotype. Nor did we have any clinical data (e.g., skin biopsy data) on patients and hence could not estimate what proportion of patients had, for instance, abnormal bruising or bleeding, etc. Further, the Swedish Patient Registry does not distinguish between various forms of EDS, and we were unable to examine psychiatric disorders and EDS divided into classical (type I) or vascular (type IV) or according to the newer Villefranche Classification of EDS [2]. Another weakness is that we only had data on EDS and hypermobility syndrome in a hospital-based setting. The Swedish Patient Registry does not record visits to general practitioners. Thus, it may be that the majority of patients with hypermobility syndrome and EDS are cared for in a non-hospital outpatient setting. At the same time, the diagnosis often requires a certain amount of investigation that is usually done in a hospital (typically with a rheumatologist) to avoid differential diagnosis misclassification. Such a work-up would likely result in a diagnosis in the Patient Registry.

We are unaware of any studies on EDS, hypermobility syndrome and ADHD. ADHD is associated with the risk of psychiatric and functional problems [24] that inevitably leads to serious consequences for the affected individuals, their families and society [24, 25]. Prevalence estimates in children (≈5 %) [26] and adults (≈2–5 %) [27] further underscore the societal impact of the disorder.

The association seen in this study may depend on several mechanisms, and not necessarily the same mechanisms for all psychiatric disorders. The disease load in hypermobility syndrome and EDS may contribute to a lower quality of life and an increased risk of depression and suicide attempt, but is less likely to explain the excess risk of other disorders such as ASD and bipolar disorder. An increased disease burden is otherwise likely to occur both before (diagnostic delay with secondary patient frustration is common in EDS) and after diagnosis (there is no curative treatment for EDS). Hypermobility syndrome, EDS and some psychiatric disorders may also share common familial risk factors. Relative to risk factors, of particular interest is our finding of an increased risk of psychiatric disorder in unaffected EDS or hypermobility syndrome siblings. This finding suggests that it is not only the disease itself that links to psychiatric disorder but also that part of the risk increase may be due to shared genetic or early environmental influences.

Among the weaknesses of this study are the limited data on clinical phenotypes such as symptoms and signs of disease of the Patient Registry. While the specificity of this register is high [14], especially for chronic disorders (including EDS or hypermobility syndrome), the sensitivity may be lower. Hence, we cannot ignore the possibility that some cases may have been missed. If such cases have less psychiatric comorbidity than individuals recorded with EDS or hypermobility in the Patient Registry, we may have overestimated the risk of psychiatric morbidity. However, considering the low prevalence of EDS or hypermobility syndrome in the general population (1/5000), false-negatives cases are unlikely to influence our estimates more than marginally.

Finally, we cannot preclude that surveillance bias in patients with EDS or hypermobility syndrome and in their siblings has contributed to the higher risks for psychiatric disorder, even though the elevated risks observed in unaffected siblings argue against a substantial effect of surveillance bias in this study.

## Conclusion

In summary, this is the largest study to date to examine the association between EDS, hypermobility syndrome and psychiatric disorders. A positive association between these disorders confirms earlier case reports and smaller studies. The relative risks for several psychiatric disorders were substantial. Because siblings without EDS or hypermobility syndrome carried an increased risk of being diagnosed with a psychiatric disorder, we suspect that these associations may be due to genetic and shared environmental factors.

## Abbreviations

ASD, autism spectrum disorder; CI, confidence interval; EDS, Ehlers-Danlos syndrome; ICD, international statistical classification of diseases and related health problems; RR, risk ratio

## Additional files

Additional file 1: Table S1. Number of patients with each included outcome by the respective registries used for identification. (DOC 32 kb) Additional file 2: Table S2. Demographic characteristics of individuals with Ehlers-Danlos syndrome (EDS) or hypermobility syndrome. (DOC 28 kb) Additional file 3: Table S3. Risks of psychiatric disorders, suicide attempt and suicide in individuals with Ehlers-Danlos syndrome (EDS) whose first EDS diagnosis preceded their first psychiatric diagnosis compared with matched comparison individuals. Associations are expressed as risk ratios (RR) and 95 % confidence intervals (95 % CIs) from conditional logistic regression. (DOC 30 kb) Additional file 4: Table S4. Risks of psychiatric disorders, suicide attempt and suicide in individuals with Ehlers-Danlos syndrome (EDS) aged ≤19 years and ≥20 years at first EDS diagnosis compared with matched comparison individuals. Associations are expressed as risk ratios (RR) and 95 % confidence intervals (95 % CIs) from conditional logistic regression. (DOC 31 kb)
