# Supplementary material for: Nationwide population-based cohort study of psychiatric disorders in individuals with Ehlers–Danlos syndrome or hypermobility syndrome and their siblings
Source: BMC Psychiatry. 2016 Jul 4;16:207. doi: 10.1186/s12888-016-0922-6 (PMC4932739; doi:10.1186/s12888-016-0922-6)
Supplement: Additional file 1: Table S1. — Number of patients with each included outcome by the respective registries used for identification. (DOC 32 kb) [file 12888_2016_922_MOESM1_ESM.doc]

**Additional file 1: Table S1.** Number of patients with each included outcome by the respective registries used for identification.

|  | National Patient Registry | Prescribed Drug Registry | Cause of Death Registry | Registry for Child and Adolescent Psychiatry in Stockholm | Overlap between Registries | Total |
| --- | --- | --- | --- | --- | --- | --- |
| Ehlers-Danlos syndrome | 1,780 | - | - | - | None | 1,780 |
| Hypermobility syndrome | 10, 019 | - | - | - | None | 10,019 |
| Autism spectrum disorder | 27,569 | - | - | 5 783 | 1 646 | 31,706 |
| Bipolar disorder | 48,180 | - | - | - | None | 48,180 |
| ADHD | 48,024 | 49,788 | - | 7 312 | 39,595 | 65,529 |
| Depression | 466,739 | - | - | - | None | 466,739 |
| Suicide attempt | 272,133 | - | - | - | None | 272,133 |
| Suicide | - | - | 78,856 | - | None | 78,856 |
| Schizophrenia | 58,334 | - | - | - | None | 58,334 |

Note: individuals with psychiatric disorders were only counted once (first date with psychiatric disorder) even if some individuals occurred in more than one registry.
