# Supplementary material for: Nationwide population-based cohort study of psychiatric disorders in individuals with Ehlers–Danlos syndrome or hypermobility syndrome and their siblings
Source: BMC Psychiatry. 2016 Jul 4;16:207. doi: 10.1186/s12888-016-0922-6 (PMC4932739; doi:10.1186/s12888-016-0922-6)
Supplement: Additional file 2: Table S2. — Demographic characteristics of individuals with Ehlers-Danlos syndrome (EDS) or hypermobility syndrome. (DOC 28 kb) [file 12888_2016_922_MOESM2_ESM.doc]

**Additional file 2: Table S2.** Demographic characteristics of individuals with Ehlers-Danlos syndrome (EDS) or hypermobility syndrome.

|  | EDS  (n=1,771) | Hypermobility syndrome  (n=10,019) |
| --- | --- | --- |
| Entry year (median, range) | 2003,1997-2009 | 2002, 1997-2009 |
| Females | 1,312 (74%) | 6,111 (67%) |
| Child (< 18years) | 618 (35%) | 4,608 (46%) |
