# Supplementary material for: Nationwide population-based cohort study of psychiatric disorders in individuals with Ehlers–Danlos syndrome or hypermobility syndrome and their siblings
Source: BMC Psychiatry. 2016 Jul 4;16:207. doi: 10.1186/s12888-016-0922-6 (PMC4932739; doi:10.1186/s12888-016-0922-6)
Supplement: Additional file 3: Table S3. — Risks of psychiatric disorders, suicide attempt and suicide in individuals with Ehlers-Danlos syndrome (EDS) whose first EDS diagnosis preceded their first psychiatric diagnosis compared with matched comparison individuals. Associations are expressed as risk ratios (RR) and 95 % confidence intervals (95 % CIs) from conditional logistic regression. (DOC 30 kb) [file 12888_2016_922_MOESM3_ESM.doc]

**Additional file 3: Table S3.** Risks of psychiatric disorders, suicide attempt and suicide in individuals with Ehlers-Danlos syndrome (EDS) whose first EDS diagnosis preceded their first psychiatric diagnosis compared with matched comparison individuals. Associations are expressed as risk ratios (RR) and 95% confidence intervals (95%CIs) from conditional logistic regression.

|  | EDS individuals  (n=1,557) | Matched comparison individuals  (n=15,570) | RR (95%CI) |
| --- | --- | --- | --- |
| Autism spectrum  disorder | 31 (2.0) | 69 (0.4) | **4.6 (3.0-7.0)** |
| Bipolar disorder | 3 (0.2) | 64 (0.4) | 0.5 (0.1-1.5) |
| ADHD | 30 (1.9) | 169 (1.1) | **1.8 (1.2-2.7)** |
| Depression | 63 (4.1) | 437 (3.4) | 1.2 (0.9-1.6) |
| Suicide attempt | 23 (1.5) | 368 (2.64) | **0.6 (0.4-0.9)** |
| Suicide | 1 | 6 (0.1) | n/a |
| Schizophrenia | 0 | 33 (0.2) | n/a |

Note: statistically significant RRs are bolded. n/a, not applicable.
