# Supplementary material for: Nationwide population-based cohort study of psychiatric disorders in individuals with Ehlers–Danlos syndrome or hypermobility syndrome and their siblings
Source: BMC Psychiatry. 2016 Jul 4;16:207. doi: 10.1186/s12888-016-0922-6 (PMC4932739; doi:10.1186/s12888-016-0922-6)
Supplement: Additional file 4: Table S4. — Risks of psychiatric disorders, suicide attempt and suicide in individuals with Ehlers-Danlos syndrome (EDS) aged ≤19 years and ≥20 years at first EDS diagnosis compared with matched comparison individuals. Associations are expressed as risk ratios (RR) and 95 % confidence intervals (95 % CIs) from conditional logistic regression. (DOC 31 kb) [file 12888_2016_922_MOESM4_ESM.doc]

**Additional file 4: Table S4.** Risks of psychiatric disorders, suicide attempt and suicide in individuals with Ehlers-Danlos syndrome (EDS) aged ≤19 years and ≥20 years at first EDS diagnosis compared with matched comparison individuals. Associations are expressed as risk ratios (RRs) and 95% confidence intervals (95%CIs) from conditional logistic regression.

|  | EDS individuals ≤19 years at first diagnosis | EDS individuals ≥20 years at first diagnosis |
| --- | --- | --- |
|  | RR (95%CI) | RR (95%CI) |
| Autism spectrum  disorder | **9.4 (3.7-28.8)** | **4.9 (2.4-9.8)** |
| Bipolar disorder | n/a | **2.6 (1.4-4.8)** |
| ADHD | **5.3 (2.0-14.6)** | **5.5 (3.3-9.1)** |
| Depression | n/a | **3.6 (3.0-4.3)** |
| Suicide attempt | 0.6 (0.1-4.9) | **2.3 (1.8-3.0)** |
| Suicide | n/a | 5.0 (0.9-27.3) |
| Schizophrenia | n/a | 0.5 (0.1-2.2) |

Note: statistically significant RRs are bolded. n/a, not applicable due to insufficient power or non-converging models. n/a, not applicable.
